# Knowledge, attitudes, and practices of adult patients with myopia toward refractive surgery and postoperative medications: a cross-sectional study

**DOI:** 10.3389/fmed.2026.1759008

**Published:** 2026-02-23

**Authors:** Huan Wei, Libin Zhou, Yangli Huang, Yanqiong Liu, Lili Sun

**Affiliations:** Department of Ophthalmology, Affiliated Hospital of Panzhihua University, Panzhihua, China

**Keywords:** attitude, knowledge, myopic refractive surgery, postoperative visual quality management, practice

## Abstract

**Introduction:**

This study aims to assess the knowledge, attitudes, and practices (KAP) of adult patients with myopia regarding refractive surgery and the use of postoperative medications.

**Methods:**

A cross-sectional survey was conducted between July 2024 and November 2024 at The Affiliated Hospital of Panzhihua University. Data were collected using a structured questionnaire.

**Results:**

A total of 433 valid responses were collected. Among the participants, 231 (53.3%) were female, 216 (49.9%) had a spherical equivalent refractive error between −3.00 and −6.00 diopters in both eyes, and 154 (35.6%) reported daily screen time ranging from 6 to 12 h. The mean (SD) knowledge, attitude, and practice scores were 10.60 (range: 0–18), 38.61 (range: 11–55), and 18.81 (range: 6–30), respectively, where the ranges indicate the minimum and maximum achievable scores. Significant positive correlations were found among knowledge, attitude and practice (*p* < 0.001). Multivariate analysis revealed that knowledge significantly influenced both attitude (OR = 1.200, 95% CI: [1.133,1.272], *p* < 0.001) and behavioral engagement (OR = 1.255, 95% CI: [1.177,1.339], p < 0.001). Structural equation modeling (SEM) results showed that knowledge directly affected attitude (*β* = 0.546, *p* < 0.001) and practice (*β* = 0.246, *p* < 0.001), attitude also directly affected practice (*β* = 0.468, *p* < 0.001). Additionally, knowledge indirectly affected practice through attitude (*β* = 0.256, *p* < 0.001).

**Conclusion:**

Patients with myopia demonstrated insufficient knowledge and suboptimal practices despite generally positive attitudes toward refractive surgery and postoperative care. These findings highlight the urgent need for structured patient education strategies to improve adherence and optimize postoperative visual quality.

## Introduction

Myopia, commonly known as nearsightedness, represents a significant and escalating global public health challenge. As of 2020, approximately 30% of the world’s population was affected by myopia, with projections indicating this proportion could increase to nearly half (49.8%) of the global population - equivalent to approximately 4.76 billion people - by 2050 (1). Uncorrected refractive errors, particularly myopia, rank among the leading causes of visual impairment and blindness worldwide (2). The prevalence is especially concerning in certain urban areas of China, where over 80% of 18-year-olds are affected by myopia (3). Given this increasing burden, the development and implementation of effective management strategies have become crucial. Standard corrective approaches for myopia include spectacles, contact lenses, and refractive surgery, each with varying costs and accessibility (4). Among surgical interventions, laser refractive procedures such as small incision lenticule extraction (SMILE), laser-assisted *in situ* keratomileusis (LASIK) and photorefractive keratectomy (PRK), have gained widespread adoption due to their demonstrated long-term cost-effectiveness in adults, particularly in developed countries (4, 5). For individuals with high myopia who may not be suitable candidates for corneal laser surgery, implantable collamer lens (ICL) implantation has emerged as a viable alternative (6). These refractive surgical procedures represent the primary surgical modalities addressed in the present study. Recent studies have demonstrated that surgical interventions can significantly improve quality of life and visual satisfaction for patients with high myopia, with ICL implantation showing superior performance in night vision, contrast sensitivity, and uncorrected distance visual acuity compared to traditional corrective methods (7). Long-term follow-up studies have shown that ICL surgery maintains stable and effective outcomes over a 12-year period, with significantly reduced risks of retinal detachment and glaucoma compared to clear lens exchange (8).

Despite the success of these surgical interventions in providing independence from corrective lenses, patients may experience postoperative complications that affect visual quality, including glare, decreased night vision, and dry eye syndrome (9). To address these adverse effects, researchers have explored various therapeutic strategies, including the use of diquafosol sodium and cyclosporine (10). In the context of this study, postoperative visual quality management refers to patient-reported symptoms and functional discomfort commonly experienced after refractive surgery, such as dry eye sensation, glare, and reduced night vision, rather than objective visual acuity outcomes. While substantial research has focused on refining surgical techniques for myopia correction, comparatively limited attention has been directed toward understanding patients’ cognitive perceptions and behavioral patterns regarding surgical interventions and postoperative care. Several clinical trials have demonstrated the efficacy of postoperative medications in managing surgical complications: diquafosol sodium has shown significant improvement in tear film stability and dry eye symptoms after LASIK surgery (11), while topical cyclosporine has effectively reduced inflammatory responses and enhanced visual quality in post-surgical patients (12). A recent meta-analysis reported that combination therapy with diquafosol and cyclosporine achieved superior outcomes in alleviating postoperative dry eye and improving contrast sensitivity compared to conventional artificial tears (10). This knowledge gap is clinically significant, as insufficient health literacy, attitudinal barriers, and non-adherence to postoperative protocols among patients may create disparities in clinical outcomes, potentially compromising long-term visual rehabilitation and refractive stability (13). Unlike adolescents, adult patients typically rely more heavily on their own knowledge and physician recommendations when making surgical decisions (14). Their knowledge, attitudes, and practices may directly impact postoperative experiences, influencing both satisfaction levels and long-term visual outcomes (15).

The Knowledge, Attitude, and Practices (KAP) survey serves as a valuable diagnostic research tool that illuminates a group’s comprehension, beliefs, and actions regarding a specific subject. This approach is particularly relevant within the realm of health literacy, as it is predicated on the premise that knowledge positively influences attitudes, which subsequently shape behaviors (16–18). In ophthalmology, previous studies have investigated KAP in relation to myopia prevention among adolescents; however, limited research has examined adult patients undergoing refractive surgery and their medication adherence and postoperative care-related treatment-seeking behaviors (19, 20).

Adult patients often report high expectations regarding surgical outcomes (21, 22). However, variability in preoperative knowledge levels and postoperative management behaviors has been observed across patient populations (22). Differences in understanding surgical procedures and postoperative care requirements may be associated with variations in treatment adherence and postoperative experiences (23). Therefore, assessing KAP in this population is essential for improving postoperative education, enhancing treatment adherence, and optimizing visual quality. This study aims to investigate the knowledge, attitudes, and practices of adult patients with myopia regarding refractive surgery and the use of postoperative medications. To our knowledge, this is the first study to apply a structural equation modeling framework to examine the interrelationships between knowledge, attitudes, and practices regarding refractive surgery and postoperative medication use in adult myopia patients.

## Materials and methods

### Study design and participants

This cross-sectional study was conducted between July and November 2024 at The Affiliated Hospital of Panzhihua University, focusing on adult patients with myopia. Participants were not required to have undergone any specific type of refractive surgery to be eligible for inclusion. Ethical approval was granted by the Ethics Committee of Panzhihua Traditional Chinese and Western Medicine Hospital (Ethics approval No. 2024-06-007), and all participants provided informed consent before participation. Eligible participants were adults aged 18 years or older with a confirmed diagnosis of myopia, defined as a spherical equivalent refractive error of ≤ − 0.50 diopters before refractive surgery, who were able to understand the questionnaire content and provided informed consent. Exclusion criteria included incomplete questionnaire responses, a questionnaire completion time of less than 60 s, or incorrect responses to quality control questions, such as reporting no history of myopia. For refractive error classification, participants were categorized using the average spherical equivalent of both eyes. Moderate myopia was defined as a spherical equivalent between −3.00 and −6.00 diopters in either eye. In this study, refractive error severity was expressed using spherical equivalent values in diopters. Postoperative medications mainly referred to commonly prescribed ocular surface treatments after refractive surgery, including diquafosol sodium eye drops and topical cyclosporine, which were specifically included in the questionnaire items assessing postoperative medication awareness and usage.

Data were collected using a structured self-administered questionnaire distributed via Wenjuanxing (Changsha Ranxing Information Technology Co., Ltd., Hunan, China), a widely used online survey platform in China, with access provided through a QR code. Participants could complete the survey through two primary methods: online distribution via WeChat groups or in-person scanning of printed QR codes available at the specialized ophthalmology outpatient clinics for myopia management, strabismus/amblyopia, and refractive surgery. Specifically, participants were recruited through two channels. First, patients who visited the hospital and met the inclusion criteria were informed about the study by trained research staff. Those who agreed to participate scanned the questionnaire QR code on site and completed the survey independently. Second, a recruitment announcement was posted in the consultation group of the refractive myopia clinic. Eligible individuals who expressed willingness to participate accessed the questionnaire via the provided QR code and completed it online. Before accessing the questionnaire, all participants were presented with an electronic informed consent form and were required to provide consent prior to proceeding with the survey. A convenience sampling approach was used. All patients who met the inclusion criteria and were willing to participate during the study period were invited to complete the questionnaire, without targeted selection of preoperative candidates or postoperative patients. Therefore, both preoperative candidates considering refractive surgery and postoperative patients attending follow-up visits were included in the study sample. To ensure data integrity, each IP address was restricted to a single submission, and all survey items were mandatory.

### Questionnaire development and pilot testing

The questionnaire was newly developed by the research team for this study and had not been previously validated or used in other studies. It was originally designed in Chinese and refined based on feedback from two senior specialists in the relevant field (both chief physicians from the department), followed by a small-scale pilot test conducted in the same setting and using the same inclusion and screening criteria as the formal study. A total of 71 questionnaires were distributed and returned; responses were then screened for validity by excluding those without informed consent, with incorrect answers to attention-check items, or with an excessively short completion time, resulting in 51 valid questionnaires included in the final pilot analysis. As the questionnaire was originally developed in Chinese, no translation or back-translation procedures were required. Reliability analysis demonstrated satisfactory internal consistency, with an overall Cronbach’s *α* coefficient of 0.882, while the subscale coefficients for the knowledge, attitude, and practice sections were 0.788, 0.807, and 0.860, respectively, indicating good reliability (See Supplementary material Questionnaire, which includes representative example items for each KAP domain). The pre-testing process included expert review for content clarity and relevance, as well as pilot participant feedback to identify ambiguous or unclear items before finalizing the questionnaire.

The final version of the questionnaire, administered in Chinese, comprised 39 items distributed across four dimensions. Demographic information included 11 items, while the knowledge section contained 10 items, with the last item serving as a trap question to identify and exclude non-myopic individuals. Participant characteristics, including academic status, average daily screen time, and use of glasses or contact lenses, were self-reported and collected as part of the demographic section of the questionnaire. In this study, “eye exercises” referred to the standardized Chinese school-based eye exercise program that has been nationally implemented since 1972 and incorporated into the Comprehensive Plan for the Prevention and Control of Myopia in Children and Adolescents (2018). This program is based on traditional Chinese massage and meridian theory and consists of a structured sequence of periocular acupoint massage intended to relieve visual fatigue and improve local blood circulation and ocular muscle tension. Participants were asked whether they had a regular habit of performing this standardized eye exercise program. The attitude section consisted of 11 items, and the practice section comprised 6 items. For statistical analysis, scoring was assigned according to the response options for each item. All item scoring rules were predefined before data collection and applied uniformly across participants to ensure consistency in domain score calculation. In the knowledge section, responses were scored as follows: “very familiar” received 2 points, “heard of” was assigned 1 point, and “unclear” received 0 points, with total scores ranging from 0 to 18. The attitude dimension included both positively and negatively worded items. Positive attitude items (A1-A4, A7, A10-A11) were rated on a 5-point Likert scale from “strongly agree” (5 points) to “strongly disagree” (1 point), while negatively worded items (A5-A6, A8-A9) were reverse-scored. The total score for attitude ranged from 11 to 55. The practice dimension exclusively contained positive practice items, also rated on a 5-point scale from “always” (5 points) to “never” (1 point), with total scores ranging from 6 to 30. These ranges represent the minimum and maximum achievable scores for each domain based on the questionnaire scoring system. A score exceeding 70% of the maximum possible score in each section was considered indicative of adequate knowledge, a positive attitude, and proactive engagement in myopia-related practices (24). The 70% cut-off was selected based on prior KAP literature to distinguish adequate from inadequate levels while preserving statistical power.

### Sample size calculation

The sample size was determined using the standard formula for cross-sectional studies, $n={\left(\frac{Z_{1-\alpha /2}}{\delta }\right)}^{2}\times p\times (1-p)$ , with a significance level (*α*) set at 0.05, corresponding to a Z-value of 1.96, an acceptable margin of error (*δ*) of 0.05, and an assumed prevalence (p) of 0.5. Based on these parameters, the minimum required sample size was calculated to be 384. Considering an anticipated effective questionnaire recovery rate of 80%, the final target was adjusted to a minimum of 480 distributed questionnaires to ensure an adequate number of valid responses for analysis.

### Statistical analysis

Statistical analyses were performed using R 4.3.2 (R Foundation for Statistical Computing, Vienna, Austria). Prior to formal analysis, data were screened for completeness and logical consistency, and no missing values were present because all questionnaire items were set as mandatory. The normality of continuous variables was assessed using a Shapiro–Wilk test. Homogeneity of variance was evaluated using Levene’s test where applicable to ensure the appropriateness of parametric comparisons. Normally distributed data were expressed as mean and standard deviation (SD), whereas non-normally distributed data were presented as median with interquartile range (IQR; 25th and 75th percentiles). Categorical variables were reported as frequency and percentage (n, %). Comparisons of dimensional scores across demographic subgroups were conducted as follows: for continuous variables, an independent t-test was used for two-group comparisons if the data were normally distributed, while the Wilcoxon-Mann–Whitney test was applied for non-normally distributed data. For comparisons among three or more groups, one-way analysis of variance (ANOVA) was used when normality and homogeneity of variance assumptions were met; otherwise, the Kruskal-Wallis test was employed. Correlations between the dimensional scores were assessed using Pearson correlation coefficients for normally distributed data and Spearman correlation coefficients for non-normally distributed data. To explore the association between demographic characteristics and each dimension’s score, univariate and multivariate logistic regression analyses were performed, with each dimension’s total score dichotomized using a 70% threshold. This approach was adopted to facilitate clinical interpretability by categorizing participants into adequate versus inadequate knowledge, positive versus negative attitude, and proactive versus non-proactive practice groups, which is commonly applied in KAP-based epidemiological studies. Variables with *p* < 0.10 in univariate analysis were incorporated into the multivariate model. The initial selection of candidate variables was determined during the study design stage based on relevant literature review and expert consultation. Potential confounding variables, including age, education level, degree of myopia (spherical equivalent), employment status, income level, screen time, use of glasses or contact lenses, and eye exercise habits, were considered during the univariate screening process, and those meeting the predefined statistical criteria were further entered into the multivariate regression models. Age and gender were not included in the final multivariate models when they did not meet the predefined significance threshold in the univariate analysis. Odds ratios (OR) with 95% confidence intervals (CI) were reported, and *p*-values were rounded to three decimal places, with *p* < 0.05 considered statistically significant. Structural equation modeling (SEM) was conducted based on the knowledge-attitude-practice (KAP) framework to test the following hypothesized pathways: (1) knowledge would affect practice both directly and indirectly through attitude; (2) knowledge would directly affect attitude; and (3) attitude would directly affect practice. Both direct and indirect effects were estimated and compared. Model fit was evaluated using the root mean square error of approximation (RMSEA), standardized root mean square residual (SRMR), Tucker-Lewis index (TLI), and comparative fit index (CFI), with acceptable fit criteria set at RMSEA < 0.08, SRMR < 0.08, TLI > 0.80, and CFI > 0.80. If the model did not meet the predefined goodness-of-fit thresholds, mediation analysis was performed using path analysis. Structural equation modeling was conducted using Stata 18.0 (StataCorp, College Station, TX, USA). Maximum likelihood estimation was applied for parameter estimation in the SEM analysis.

## Results

Initially, a total of 518 samples were collected. The following cases were excluded: 17 cases with response times shorter than 60 s; 5 cases with abnormal age entries; 63 cases failing the quality control question (indicating non-myopic individuals); Ultimately, 433 valid datasets were obtained, with an effective rate of 83.59%. In the formal experiment, the overall scale and subscales demonstrated good internal consistency, with an overall Cronbach’s *α* coefficient of 0.9388. The Cronbach’s α coefficients for the knowledge, attitude, and practice sections were 0.8776, 0.8965, and 0.9251, respectively. The Kaiser-Meyer-Olkin (KMO) value for the overall scale was 0.9224.

### Demographic characteristics of participants

Among the participants, 231 (53.3%) were female, 241 (55.7%) were aged 18–24 years old, 283 (65.4%) lived in urban areas, 365 (84.3%) had a bachelor’s degree or above, 216 (49.9%) had a spherical equivalent refractive error between −3.00 and −6.00 diopters in both eyes, 154 (35.6%) had daily screen time of 6–12 h, 333 (76.9%) spent less than 30 min on daily eye relaxation exercises, and 328 (75.8%) wore glasses. The mean (SD) knowledge, attitude, and practice scores were 10.60 (range: 0–18), 38.61 (range: 11–55), and 18.81 (range: 6–30), respectively, where the ranges indicate the minimum and maximum achievable scores. Knowledge scores varied significantly by residence, mean spherical equivalent refractive error in both eyes (diopters), and habit of doing eye exercises (all *p* < 0.05). Attitude scores varied significantly by monthly income per capita, mean spherical equivalent refractive error in both eyes, and whether wearing glasses (all *p* < 0.05). Practice scores varied significantly by age, education, current employment status, daily screen time, and habit of doing eye exercises (all *p* < 0.05) (Table 1).

**Table 1:** Demographic characteristics and knowledge, attitude, and practice scores of adult myopia patients regarding refractive surgery and postoperative medication use (*N* = 433).

*N* = 433	*N* (%)	Knowledge	*p*	Attitude	*p*	Practice	*p*
		Mean (SD)		Mean (SD)		Mean (SD)	
Total score	433 (100.0)	10.60 (3.96)		38.61 (3.54)		18.81 (5.72)	
Gender			0.383		0.190		0.599
Male	202 (46.7)	10.43 (4.14)		38.35 (3.41)		19.06 (6.11)	
Female	231 (53.3)	10.75 (3.79)		38.84 (3.64)		18.58 (5.36)	
Age			0.667		0.067		0.016
18–24	241 (55.7)	10.71 (3.64)		38.55 (3.57)		19.48 (5.64)	
25–34	89 (20.6)	10.66 (4.18)		39.26 (3.57)		18.60 (6.02)	
35 or more	103 (23.8)	10.29 (4.47)		38.20 (3.41)		17.42 (5.42)	
Residence			0.022		0.196		0.977
Urban	283 (65.4)	10.85 (3.95)		38.80 (3.53)		18.74 (5.67)	
Rural/suburban	150 (34.6)	10.13 (3.94)		38.27 (3.54)		18.93 (5.83)	
Education			0.891		0.412		0.011
High school or below	68 (15.7)	10.57 (3.65)		38.97 (3.43)		20.24 (5.26)	
Bachelor’s degree or above	365 (84.3)	10.61 (4.02)		38.55 (3.56)		18.54 (5.77)	
Current employment status			0.607		0.965		0.032
Employed	218 (50.3)	10.74 (4.25)		38.61 (3.55)		18.22 (5.85)	
Unemployed/retired	52 (12.1)	10.98 (4.28)		38.63 (3.40)		19.48 (5.75)	
Other	163 (37.6)	10.29 (3.42)		38.60 (3.60)		19.37 (5.49)	
Monthly income per capita			0.077		0.038		0.646
<2000	48 (11.1)	11.29 (4.66)		37.88 (3.72)		19.77 (6.75)	
2000–5,000	161 (37.2)	10.06 (3.82)		38.11 (3.01)		18.71 (5.97)	
5,000–10,000	138 (31.9)	10.52 (3.89)		38.92 (3.77)		18.82 (5.40)	
10,000–20,000	86 (19.8)	11.35 (3.80)		39.48 (3.80)		18.42 (5.15)	
Average spherical equivalent refractive error in both eyes (diopters)			0.018		0.016		0.603
< −3.00 D	154 (35.6)	10.47 (4.44)		37.92 (3.38)		18.66 (5.90)	
−3.00 to −6.00 D	216 (49.9)	10.31 (3.56)		38.88 (3.49)		18.70 (5.60)	
> − 6.00 D	63 (14.5)	11.89 (3.83)		39.40 (3.85)		19.52 (5.73)	
Daily screen time			0.736		0.893		0.002
Less than 1 h	102 (23.6)	10.78 (4.35)		38.43 (3.65)		19.95 (6.19)	
4–6 h	146 (33.7)	10.84 (3.60)		38.58 (3.30)		19.40 (5.13)	
6–12 h	154 (35.6)	10.23 (3.83)		38.75 (3.73)		17.71 (5.52)	
More than 12 h	31 (7.2)	10.74 (4.84)		38.68 (3.40)		17.65 (6.79)	
Daily time spent on eye relaxation exercises			0.536		0.610		0.234
Less than 30 min	333 (76.9)	10.50 (3.96)		38.51 (3.54)		18.78 (5.81)	
30–60 min	78 (18.0)	11.00 (3.70)		38.86 (3.49)		18.51 (5.63)	
More than 60 min	22 (5.1)	10.64 (4.87)		39.32 (3.80)		20.18 (4.59)	
Habit of doing eye exercises			0.002		0.109		<0.001
No	384 (88.7)	10.39 (3.89)		38.47 (3.45)		18.32 (5.61)	
Yes	49 (11.3)	12.29 (4.11)		39.71 (4.08)		22.63 (5.13)	
Glasses (or contact lenses)			0.644		0.038		0.321
No	105 (24.2)	10.41 (4.40)		37.97 (3.77)		19.06 (5.72)	
Yes	328 (75.8)	10.66 (3.81)		38.82 (3.44)		18.73 (5.73)	

Knowledge score range: 0–18; attitude score range: 11–55; practice score range: 6–30. Values are presented as mean (SD). *p* values were calculated using independent t-test or one-way analysis of variance (ANOVA) for normally distributed variables, and Mann–Whitney U test or Kruskal-Wallis test for non-normally distributed variables, as appropriate.

### Knowledge, attitude, and practice

The distribution of knowledge dimensions showed that the three questions with the highest number of participants choosing the “Unclear” option were “You have heard that eye drops such as diquafosol sodium and cyclosporine can help alleviate eye dryness and discomfort following refractive surgery.” (K7) with 41.34%, “You have heard that for pathological myopia, in addition to laser and surgical treatments, its complications can also be treated with photodynamic therapy and medications therapy.” (K9) with 31.41%, and “You have heard that individuals with higher degrees of myopia may experience symptoms beyond poor distance vision, such as night vision impairment, floaters, floating spots, and flashes of light.” (K2) with 17.09% (Table 2).

**Table 2:** Distribution of responses to knowledge dimension items regarding refractive surgery and postoperative visual quality improvement among adult myopia patients (*N* = 433).

Knowledge	Very familiar	Heard of it	Unclear
1. You are aware of whether your myopia is classified as low, moderate, high, or extreme myopia.	212 (48.96%)	166 (38.34%)	55 (12.7%)
2. You have heard that individuals with higher degrees of myopia may experience symptoms beyond poor distance vision, such as night vision impairment, floaters, floating spots, and flashes of light.	111 (25.64%)	248 (57.27%)	74 (17.09%)
3. You have heard that close-range activities (such as reading at a short distance) are a significant risk factor for myopia progression.	175 (40.42%)	231 (53.35%)	27 (6.24%)
4. You have heard that appropriate outdoor activities can significantly reduce the likelihood of developing myopia.	156 (36.03%)	251 (57.97%)	26 (6%)
5. You have heard that proper lighting conditions and performing eye exercises in moderation can effectively relieve visual fatigue.	182 (42.03%)	232 (53.58%)	19 (4.39%)
6. You have heard that wearing properly prescribed eyeglasses, contact lenses, and undergoing surgical correction are all effective treatment methods for simple myopia.	167 (38.57%)	244 (56.35%)	22 (5.08%)
7. You have heard that eye drops such as diquafosol sodium and cyclosporine can help alleviate eye dryness and discomfort following refractive surgery.	75 (17.32%)	179 (41.34%)	179 (41.34%)
8. You have heard that surgical correction methods include laser corneal refractive surgery (such as femtosecond laser/SMILE) and intraocular lens implantation (such as ICL surgery).	118 (27.25%)	279 (64.43%)	36 (8.31%)
9. You have heard that for pathological myopia, in addition to laser and surgical treatments, its complications (such as age-related macular degeneration and choroidal neovascularization) can also be treated with photodynamic therapy and medications (such as anti-vascular endothelial growth factor (anti-VEGF) therapy).	71 (16.4%)	226 (52.19%)	136 (31.41%)

Response options for knowledge items were scored as: very familiar = 2, heard of = 1, unclear = 0.

Responses to the attitude dimension showed that nearly two-third strongly agreed or agreed that they worry about prolonged myopia may affect vision recovery and could even lead to blindness (A5), over 70% strongly agreed or agreed that they worry about myopia may recur after undergoing laser corneal surgery (A9), and 15.01% strongly agreed and 36.49% agreed that they are concerned that treatments such as diquafosol sodium and cyclosporine eye drops may have additional side effects (A8) (Table 3).

**Table 3:** Distribution of responses to Attitude dimension items regarding refractive surgery and postoperative visual quality improvement among adult myopia patients (*N* = 433).

Attitude	Strongly agree	Agree	Neutral	Disagree	Strongly disagree
1. You are very interested in learning about the causes of myopia, eye care methods, and treatment options (P).	140 (32.33%)	203 (46.88%)	87 (20.09%)	1 (0.23%)	2 (0.46%)
2. You believe that if myopia is not properly corrected and managed, it may progress to high myopia and affect daily life (P).	178 (41.11%)	212 (48.96%)	41 (9.47%)	2 (0.46%)	0 (0%)
3. You believe that the current vision correction and eye care methods you use are scientific and reliable (P).	102 (23.56%)	173 (39.95%)	138 (31.87%)	17 (3.93%)	3 (0.69%)
4. You trust the vision correction, eye care, or treatment plans provided by your doctor (P).	157 (36.26%)	227 (52.42%)	48 (11.09%)	1 (0.23%)	0 (0%)
5. You worry that prolonged myopia may affect vision recovery and could even lead to blindness (N).	104 (24.02%)	176 (40.65%)	113 (26.1%)	33 (7.62%)	7 (1.62%)
6. You believe that not paying attention to proper eye distance in daily activities is not a big concern because there are always treatments available (N).	74 (17.09%)	102 (23.56%)	60 (13.86%)	157 (36.26%)	40 (9.24%)
7. You are confident that strictly following the doctor’s treatment plan can effectively improve your vision (P).	124 (28.64%)	233 (53.81%)	68 (15.7%)	8 (1.85%)	0 (0%)
8. You are concerned that treatments such as diquafosol sodium and cyclosporine eye drops may have additional side effects (N).	65 (15.01%)	158 (36.49%)	163 (37.64%)	46 (10.62%)	1 (0.23%)
9. You worry that myopia may recur after undergoing laser corneal surgery (N).	91 (21.02%)	225 (51.96%)	103 (23.79%)	13 (3%)	1 (0.23%)
10. If you undergo future pharmacological or surgical treatments, you believe you will be able to follow medical advice on postoperative eye care and maintenance (P).	122 (28.18%)	244 (56.35%)	64 (14.78%)	2 (0.46%)	1 (0.23%)
11. If you undergo future pharmacological or surgical treatments, you believe regular follow-up examinations are essential to monitor treatment effectiveness (P).	135 (31.18%)	249 (57.51%)	47 (10.85%)	2 (0.46%)	0 (0%)

Attitude items were rated on a 5-point Likert scale from strongly agree (5) to strongly disagree (1), with negatively worded items reverse scored.

Responses to the practice dimension showed that the majority never use diquafosol sodium eye drops, cyclosporine eye drops, or other treatments to relieve eye discomfort (P2), nearly one-third rarely or never discuss with their doctor the indications and contraindications to determine the best medical decision (P6), 24.02% rarely and 4.39% never actively share correct eye care knowledge with friends and family (P5) (Table 4).

**Table 4:** Distribution of responses to practice dimension items regarding refractive surgery and postoperative visual quality improvement among adult myopia patients (*N* = 433).

Practice	Always	Often	Sometimes	Rarely	Never
1. You actively learn about proper eye protection, treatment, and related knowledge (P).	77 (17.78%)	115 (26.56%)	170 (39.26%)	68 (15.7%)	3 (0.69%)
2. You frequently use diquafosol sodium eye drops, cyclosporine eye drops, or other treatments to relieve eye discomfort (P).	53 (12.24%)	50 (11.55%)	71 (16.4%)	135 (31.18%)	124 (28.64%)
3. You actively learn about foods beneficial for vision and regularly consume them to improve eyesight (P).	69 (15.94%)	85 (19.63%)	176 (40.65%)	89 (20.55%)	14 (3.23%)
4. You actively learn about outdoor activities beneficial for vision and practice them regularly (P).	73 (16.86%)	90 (20.79%)	168 (38.8%)	91 (21.02%)	11 (2.54%)
5. You actively share correct eye care knowledge with friends and family (P).	66 (15.24%)	89 (20.55%)	155 (35.8%)	104 (24.02%)	19 (4.39%)
6. You discuss with your doctor the indications and contraindications of medications and surgical treatments for myopia to determine the best medical decision for you (P).	81 (18.71%)	81 (18.71%)	137 (31.64%)	98 (22.63%)	36 (8.31%)

Practice items were rated on a 5-point Likert scale from always (5) to never (1).

### Correlations between KAP

Correlation analysis showed that there were significant positive correlations between knowledge and attitude (r = 0.247, 95% CI: 0.156–0.333, *p* < 0.001) as well as practice (r = 0.412, 95% CI: 0.331–0.487, *p* < 0.001). In addition, a significant correlation was observed between attitude and practice (r = 0.208, 95% CI: 0.116–0.296, *p* < 0.001) (Table 5).

**Table 5:** Spearman correlation analysis of knowledge, attitude, and practice scores among adult myopia patients (*N* = 433).

Spearman	Knowledge	Attitude	Practice
Knowledge	1.000		
Attitude	0.247 (0.156, 0.333) ***	1.000	
Practice	0.412 (0.331, 0487) ***	0.208 (0.116, 0.296) ***	1.000

***p < 0.001.

### Univariate and multivariate analysis

The 70% of the highest possible scores for the knowledge, attitude, and practice dimensions were used as cut-off values to categorize participants into higher and lower performance groups, and the number of participants below the cut-off value were 312 (72.06%), 207 (47.81%), and 139 (32.1%), respectively (Supplementary Table S1). Multivariate logistic regression showed that other current employment status (OR = 0.538, 95% CI: [0.329, 0.877], *p* = 0.013) and with habit of doing eye exercises (OR = 2.263, 95% CI: [1.197, 4.277], *p* = 0.012) were independently associated with knowledge (Table 6). Concurrently, knowledge (OR = 1.200, 95% CI: [1.133, 1.272], *p* < 0.001) was independently associated with positive attitude (Table 7). Furthermore, knowledge (OR = 1.255, 95% CI: [1.177, 1.339], *p* < 0.001) and with habit of doing eye exercises (OR = 2.282, 95% CI: [1.136, 4.584], *p* = 0.020) were independently associated with proactive practice (Table 8).

**Table 6:** Univariate and multivariate logistic regression analysis of factors associated with adequate knowledge regarding refractive surgery and postoperative visual quality improvement among adult myopia patients (*N* = 433) (binary logistic regression model).

Knowledge	Univariate analysis	*p*	Multivariate analysis	*p*
	OR (95%CI)		OR (95%CI)	
Gender				
Male				
Female	1.021 (0.670, 1.558)	0.923		
Age				
18–24				
25–34	1.297 (0.756, 2.195)	0.338		
35 or more	1.161 (0.690, 1.929)	0.568		
Residence				
Urban				
Rural/suburban	0.697 (0.438, 1.092)	0.121		
Education				
High school or below				
Bachelor’s degree or above	1.092 (0.618, 2.004)	0.768		
Current employment status				
Employed				
Unemployed/retired	0.857 (0.431, 1.638)	0.649	0.747 (0.373, 1.499)	0.412
Other	0.599 (0.373, 0.951)	0.032	0.538 (0.329, 0.877)	0.013
Monthly income per capita				
<2000				
2000–5,000	0.525 (0.263, 1.069)	0.071	0.520 (0.250, 1.080)	0.080
5,000–10,000	0.744 (0.373, 1.513)	0.406	0.667 (0.320, 1.392)	0.280
10,000–20,000	0.880 (0.420, 1.871)	0.737	0.775 (0.354, 1.697)	0.523
Average spherical equivalent refractive error in both eyes (diopters)				
< −3.00 D				
−3.00 to −6.00 D	0.773 (0.483, 1.239)	0.282	0.759 (0.468, 1.230)	0.263
> − 6.00 D	1.757 (0.949, 3.237)	0.071	1.725 (0.919, 3.239)	0.090
Daily screen time				
Less than 1 h				
4–6 h	1.085 (0.616, 1.930)	0.780		
6–12 h	1.008 (0.574, 1.789)	0.978		
More than 12 h	1.754 (0.741, 4.069)	0.193		
Daily time spent on eye relaxation exercises				
Less than 30 min				
30–60 min	1.200 (0.692, 2.037)	0.507		
More than 60 min	1.260 (0.468, 3.091)	0.626		
Habit of doing eye exercises				
No				
Yes	2.130 (1.147, 3.909)	0.015	2.263 (1.197, 4.277)	0.012
Glasses (or contact lenses)				
No				
Yes	0.960 (0.594, 1.579)	0.869		

**Table 7:** Univariate and multivariate logistic regression analysis of factors associated with positive attitude regarding refractive surgery and postoperative visual quality improvement among adult myopia patients (*N* = 433) (binary logistic regression model).

Attitude	Univariate analysis	*p*	Multivariate analysis	*p*
	OR (95%CI)		OR (95%CI)	
Knowledge	1.193 (1.128, 1.261)	<0.001	1.200 (1.133, 1.272)	<0.001
Gender				
Male				
Female	1.136 (0.779, 1.659)	0.508		
Age				
18–24				
25–34	1.737 (1.058, 2.891)	0.031	1.610 (0.931, 2.785)	0.088
35 or more	0.819 (0.514, 1.299)	0.397	0.834 (0.501, 1.388)	0.485
Residence				
Urban				
Rural/suburban	0.874 (0.588, 1.299)	0.506		
Education				
High school or below				
Bachelor’s degree or above	0.900 (0.533, 1.511)	0.690		
Current employment status				
Employed				
Unemployed/retired	1.129 (0.616, 2.090)	0.695		
Other	0.907 (0.604, 1.361)	0.637		
Monthly income per capita				
<2000				
2000–5,000	1.239 (0.649, 2.389)	0.518	1.432 (0.699, 2.933)	0.327
5,000–10,000	1.531 (0.792, 2.990)	0.207	1.718 (0.824, 3.582)	0.149
10,000–20,000	1.874 (0.921, 3.861)	0.085	1.779 (0.808, 3.921)	0.153
Average spherical equivalent refractive error in both eyes (diopters)				
< −3.00 D				
−3.00 to −6.00 D	1.408 (0.930, 2.135)	0.106	1.386 (0.877, 2.190)	0.162
> − 6.00 D	1.664 (0.923, 3.033)	0.093	1.212 (0.637, 2.304)	0.558
Daily screen time				
Less than 1 h				
4–6 h	1.364 (0.822, 2.270)	0.231	1.360 (0.787, 2.350)	0.271
6–12 h	1.155 (0.700, 1.909)	0.574	1.217 (0.707, 2.094)	0.479
More than 12 h	2.046 (0.903, 4.832)	0.092	1.969 (0.784, 4.941)	0.149
Daily time spent on eye relaxation exercises				
Less than 30 min				
30–60 min	1.050 (0.641, 1.725)	0.847		
More than 60 min	1.658 (0.691, 4.248)	0.268		
Habit of doing eye exercises				
No				
Yes	1.515 (0.831, 2.824)	0.181		
Glasses (or contact lenses)				
No				
Yes	1.340 (0.863, 2.086)	0.193		

**Table 8:** Univariate and multivariate logistic regression analysis of factors associated with proactive practice regarding refractive surgery and postoperative visual quality improvement among adult myopia patients (*N* = 433) (binary logistic regression model).

Practice	Univariate analysis	*p*	Multivariate analysis	*p*
	OR (95%CI)		OR (95%CI)	
Knowledge	1.260 (1.187, 1.338)	<0.001	1.255 (1.177, 1.339)	<0.001
Attitude	1.088 (1.027, 1.152)	0.004	1.024 (0.959, 1.094)	0.476
Gender				
Male				
Female	0.912 (0.609, 1.368)	0.657		
Age				
18–24				
25–34	0.950 (0.564, 1.579)	0.846	1.069 (0.594, 1.926)	0.823
35 or more	0.599 (0.350, 1.000)	0.055	0.637 (0.352, 1.153)	0.136
Residence				
Urban				
Rural/suburban	0.947 (0.617, 1.445)	0.803		
Education				
High school or below				
Bachelor’s degree or above	0.724 (0.426, 1.251)	0.239		
Current employment status				
Employed				
Unemployed/retired	1.024 (0.520, 1.946)	0.945		
Other	1.238 (0.803, 1.909)	0.333		
Monthly income per capita				
<2000				
2000–5,000	0.814 (0.421, 1.597)	0.543		
5,000–10,000	0.645 (0.327, 1.290)	0.209		
10,000–20,000	0.557 (0.262, 1.183)	0.127		
Average spherical equivalent refractive error in both eyes (diopters)				
< −3.00 D				
−3.00 to −6.00 D	1.264 (0.807, 1.996)	0.310	1.362 (0.809, 2.291)	0.245
> − 6.00 D	1.698 (0.913, 3.139)	0.092	1.443 (0.722, 2.885)	0.299
Daily screen time				
Less than 1 h				
4–6 h	0.752 (0.445, 1.271)	0.286	0.744 (0.411, 1.347)	0.330
6–12 h	0.540 (0.316, 0.920)	0.024	0.586 (0.322, 1.068)	0.081
More than 12 h	0.518 (0.200, 1.228)	0.150	0.418 (0.149, 1.172)	0.097
Daily time spent on eye relaxation exercises				
Less than 30 min				
30–60 min	1.010 (0.588, 1.699)	0.970		
More than 60 min	1.224 (0.476, 2.948)	0.660		
Habit of doing eye exercises				
No				
Yes	3.279 (1.794, 6.081)	<0.001	2.282 (1.136, 4.584)	0.020
Glasses (or contact lenses)				
No				
Yes	0.830 (0.524, 1.327)	0.429		

### Interactions between KAP

**Figure 1:**
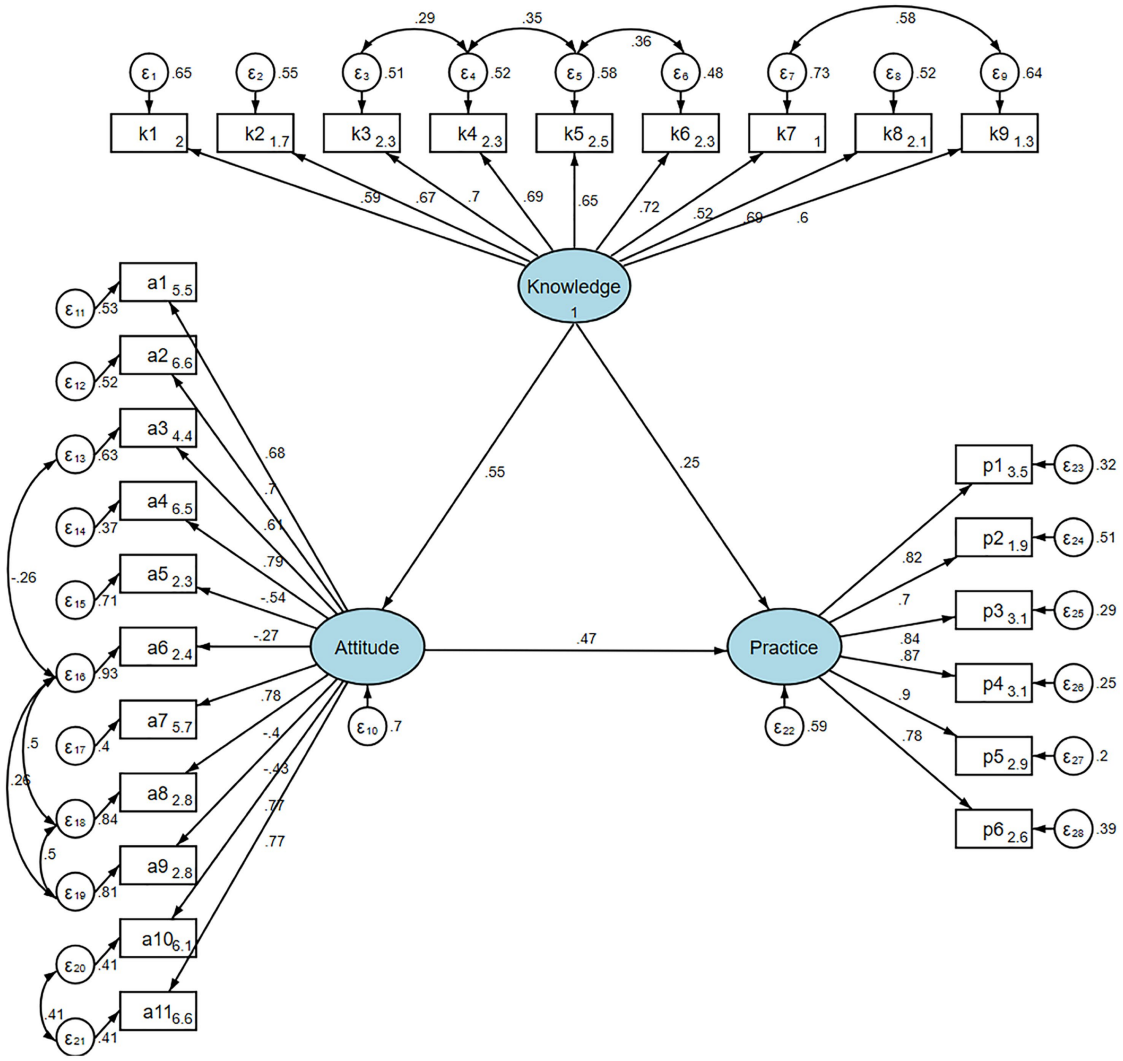
Structural equation modeling (SEM) analysis of the relationships between knowledge, attitude, and practice regarding refractive surgery and postoperative visual quality improvement among adult myopia patients (*N* = 433). The model illustrates direct and indirect pathways, with standardized path coefficients (*β*) shown for each relationship. Solid arrows represent statistically significant pathways (*p* < 0.001). Knowledge directly influences both attitude (*β* = 0.546) and practice (*β* = 0.246), while also indirectly affecting practice through attitude (*β* = 0.256). Attitude directly influences practice (*β* = 0.468).

The fit of the SEM model demonstrated acceptable goodness-of-fit, with RMSEA = 0.070, SRMR = 0.079, TLI = 0.894, and CFI = 0.906, all meeting the recommended thresholds (Supplementary Table S2), and the effect estimates between KAP were detailed in Supplementary Table S3. Analysis of the direct and indirect effects of the model showed that knowledge directly affected attitude (*β* = 0.546, *p* < 0.001) and practice (*β* = 0.246, *p* < 0.001), attitude also directly affected practice (*β* = 0.468, *p* < 0.001). Additionally, knowledge indirectly affected practice through attitude (*β* = 0.256, *p* < 0.001) (Supplementary Table S4; Figure 1).

## Discussion

Patients with myopia demonstrated insufficient knowledge, generally positive attitudes, and suboptimal engagement in practices related to refractive surgery and the use of postoperative medications. While participants generally exhibited positive attitudes, their knowledge remained inadequate, and engagement in evidence-based practices was limited. This pattern reflects broader challenges observed in ophthalmology, where gaps in patient education often led to poor adherence to treatment and follow-up care (25, 26). The results further align with studies suggesting that individuals with inadequate knowledge tend to delay seeking medical intervention or fail to adhere to prescribed postoperative care protocols, potentially leading to poorer visual outcomes (27, 28). This pattern was particularly evident in specific knowledge dimensions, where substantial proportions of participants reported being unclear about key aspects of myopia management. Notably, 41.34% of participants were unfamiliar with the use of eye drops such as diquafosol sodium and cyclosporine for alleviating post-surgical eye dryness (K7), while 31.41% lacked awareness of treatment options for pathological myopia complications (K9). Similarly, in the practice dimension, 59.82% of participants reported ‘Rarely’ or ‘Never’ using medications like diquafosol sodium or cyclosporine eye drops for eye discomfort relief (P2), and 30.94% ‘Rarely’ or ‘Never’ consulted with doctors about treatment indications and contraindications (P6). These findings highlight priority areas for targeted educational interventions to improve postoperative management and adherence.

Correlation analyses reinforce these observations, demonstrating positive associations between knowledge and both attitude and practice. SEM further supports this hierarchical relationship, indicating that knowledge not only has a direct impact on attitude and practice but also exerts an indirect effect on practice through attitude. Although SEM provides insight into directional relationships, causal inferences cannot be drawn due to the cross-sectional design. Similar KAP patterns have been reported in international ophthalmic studies. Previous surveys conducted in refractive surgery populations and eye care settings have shown that higher levels of patient knowledge are consistently associated with better treatment adherence and more favorable health behaviors, while gaps in postoperative education are linked to suboptimal compliance and follow-up attendance. Studies from different regions have also highlighted that insufficient understanding of postoperative care protocols remains a common challenge across healthcare systems, underscoring the global relevance of improving patient-centered education strategies in refractive and ophthalmic care. This pattern aligns with established theoretical frameworks in health behavior research, which emphasize the role of knowledge as a fundamental driver of attitude formation and subsequent behavioral change (29, 30). However, multiple barriers, including psychological factors, healthcare accessibility, and misinformation, may hinder effective translation of knowledge into practice (31, 32). Limited access to structured postoperative education, concerns about medication safety and cost, time constraints during outpatient consultations, and reliance on informal online information sources may hinder effective knowledge acquisition and behavioral implementation. To address these issues, standardized preoperative counseling, clearer postoperative education materials, and the use of digital health tools such as mobile reminders and brief educational modules may help improve patient understanding, engagement, and adherence to recommended care practices.

Multivariate logistic regression further clarifies the determinants of KAP. Employment status emerged as a significant predictor of knowledge, with individuals in alternative employment situations, such as students or part-time workers, demonstrating lower knowledge levels. This finding is consistent with prior research indicating that individuals with structured employment schedules often have greater exposure to health-related information, possibly through workplace wellness programs or employer-sponsored health screenings (31, 32). Another key predictor of knowledge was engagement in eye exercises, suggesting that individuals who actively participate in preventive practices are also more likely to seek out and retain information related to myopia management. However, it should be noted that the eye exercise variable in this study referred specifically to the standardized Chinese school-based eye exercise program rather than individualized ocular training regimens. Although this program is widely implemented in China, its effectiveness in improving refractive outcomes remains controversial, and adherence and execution quality may vary substantially among individuals. Therefore, the observed associations should be interpreted with caution, and future studies incorporating objective adherence measures and standardized performance assessments are warranted.

Knowledge also played a central role in shaping attitudes, with regression analysis confirming that higher knowledge levels were associated with more positive attitudes toward myopia treatment and management. However, other demographic factors, such as education and income, did not significantly influence attitudes, contrasting with prior research suggesting that higher educational attainment is linked to greater health awareness and proactive decision-making (33). This discrepancy may reflect the relatively high educational background of the study sample, which may have minimized knowledge differences across educational strata.

The relationship between attitude and practice was less pronounced, with no significant association observed in the regression model. This finding highlights the knowledge-practice gap documented in health behavior research, where positive attitudes alone are often insufficient to drive behavioral change (20). Factors such as perceived treatment efficacy, financial constraints, and accessibility to medical care likely influence practice engagement independently of attitudes. Notably, individuals who regularly performed eye exercises were more likely to adopt recommended eye care practices, suggesting that habitual engagement in one aspect of ocular health may foster greater overall adherence to best practices.

Examining the distribution of KAP scores across different demographic groups reveals additional disparities. While gender differences were not significant, younger participants reported more active engagement in practice-related behaviors. This finding aligns with broader healthcare trends, where younger individuals, often more digitally connected, are more likely to access and act upon health information through online platforms and social media (34, 35). Urban residents also demonstrated higher knowledge scores than their rural counterparts, reflecting the well-documented disparities in healthcare access and educational outreach between urban and rural populations (36).

The severity of myopia was another notable factor influencing KAP. Participants with higher degrees of myopia exhibited greater knowledge and more positive attitudes, likely due to increased exposure to medical professionals and treatment-related information. However, this increased awareness did not necessarily translate into more proactive practices, suggesting that knowledge alone is not sufficient to drive behavior change. This finding is consistent with prior research on chronic disease management, where patients with severe conditions often exhibit high levels of awareness but do not always engage in optimal self-care behaviors due to psychological distress, skepticism toward medical recommendations, or perceived treatment burdens (37).

Given the substantial gaps identified in this study, several targeted interventions are warranted. First, improving patient education should be a priority, particularly for individuals in non-traditional employment settings and those with lower baseline knowledge levels. Digital health tools, including mobile applications and interactive e-learning platforms, could be leveraged to deliver personalized, engaging, and accessible information tailored to different population groups (38).

Additionally, healthcare providers should take a more active role in reinforcing behavioral change through structured counseling and follow-up interventions. Incorporating brief educational sessions into routine eye exams, particularly for individuals undergoing surgical treatment, may help bridge the gap between knowledge and practice. Similarly, ensuring that postoperative care instructions are clear, actionable, and reinforced through multiple communication channels, including printed materials and follow-up phone calls, may enhance adherence to recommended practices (39).

At a broader policy level, targeted outreach initiatives aimed at rural populations could help address the observed disparities in knowledge and access to care. Community-based health education programs, possibly integrated into local schools or workplaces, may serve as an effective means of disseminating critical information about myopia management (40). Given the significant influence of digital screen time on practice behaviors, public health campaigns should also focus on promoting healthier screen usage habits (41). From a practical perspective, these findings have implications for clinicians and healthcare administrators. For surgeons, incorporating structured preoperative education and standardized postoperative medication counseling into routine clinical workflows may help improve patient understanding and adherence. For hospital managers and policymakers, developing unified educational materials and promoting digital follow-up systems could enhance continuity of care and patient engagement. In patient counseling, emphasizing individualized guidance based on patients’ refractive status and treatment stage may further support informed decision-making and appropriate postoperative care behaviors.

This study has several limitations. First, the single-center design and single-country cultural context may limit the generalizability of the findings to other healthcare systems and sociocultural settings. Second, the cross-sectional nature of the study precludes causal inference regarding the relationships among knowledge, attitudes, and practices. Third, reliance on self-reported questionnaire data and online survey administration may introduce recall bias, social desirability bias, and selection bias related to digital access and participation behavior, potentially affecting response accuracy. Fourth, prior surgical history was not collected as a study variable, which precluded subgroup analyses comparing knowledge, attitudes, and practices between participants with and without previous ocular or refractive surgery. Finally, objective postoperative clinical outcome measures, such as visual acuity or contrast sensitivity, were not collected, which limits the ability to directly link patient-reported practices with actual optimize postoperative visual quality.

## Conclusion

In conclusion, patients with myopia demonstrated insufficient knowledge yet generally positive attitudes, but their actual practices regarding refractive surgery and the use of postoperative medications remained suboptimal. Integrating structured educational interventions into refractive surgery pathways may represent a cost-effective strategy to enhance patient engagement, medication adherence, and long-term visual quality.

## Data Availability

The original contributions presented in the study are included in the article/Supplementary material, further inquiries can be directed to the corresponding author.
