# Supplementary material for: Knowledge, attitudes, and practices of adult patients with myopia toward refractive surgery and postoperative medications: a cross-sectional study
Source: Front Med (Lausanne). 2026 Feb 23;13:1759008. doi: 10.3389/fmed.2026.1759008 (PMC12967940; doi:10.3389/fmed.2026.1759008)
Supplement: Supplementary file 1 [file Data_Sheet_1.docx]

**Supplementary Table 1 Distribution of participants based on cutoff values (70% of total score) for Knowledge, Attitude, and Practice dimensions regarding refractive surgery and postoperative visual quality improvement (N=433)**

| **Cutoff value: 70% of the total score** | N (%) |
| --- | --- |
| Knowledge total score |  |
| Ksum>=13 | 121(27.94%) |
| Ksum<=12 | 312(72.06%) |
| Attitude total score |  |
| Asum>=38 | 226(52.19%) |
| Asum<=37 | 207(47.81%) |
| Practice total score |  |
| Psum>=21 | 294(67.9%) |
| Psum<=20 | 139(32.1%) |

**Supplementary Table 2 Goodness-of-fit indices for structural equation modeling of Knowledge, Attitude, and Practice regarding refractive surgery and postoperative visual quality improvement**

| **Indicators** | **Reference** | **Results** |
| --- | --- | --- |
| RMSEA | <0.08 | 0.070 |
| SRMR | <0.08 | 0.079 |
| TLI | >0.80 | 0.894 |
| CFI | >0.80 | 0.906 |

**Supplementary Table 3 Path analysis results from structural equation modeling examining relationships between Knowledge, Attitude, and Practice regarding refractive surgery and postoperative visual quality improvement**

| **Indicators** |  | **Estimate** | **P>\|z\|** |
| --- | --- | --- | --- |
| Attitude |  |  |  |
|  | Knowledge | 13.16 | <0.001 |
| Practice |  |  |  |
|  | Knowledge | 9.22 | <0.001 |
|  | Attitude | 11.68 | <0.001 |

**Supplementary Table 4 Direct, indirect, and total effects from structural equation modeling examining relationships between Knowledge, Attitude, and Practice regarding refractive surgery and postoperative visual quality improvement**

| **Model paths** | | Total effects | |  | Direct Effect | |  | Indirect effect | |
| --- | --- | --- | --- | --- | --- | --- | --- | --- | --- |
|  |  | β(95%CI) | P |  | β(95%CI) | P |  | β(95%CI) | P |
| Attitude |  |  |  |  |  |  |  |  |  |
|  | Knowledge | 0.546 (0.465, 0.627) | <0.001 |  | 0.546 (0.465, 0.627) | <0.001 |  |  |  |
| Practice |  |  |  |  |  |  |  |  |  |
|  | Knowledge | 0.502 (0.418, 0.586) | <0.001 |  | 0.246 (0.138, 0.354) | <0.001 |  | 0.256 (0.189, 0.322) | <0.001 |
|  | Attitude | 0.468 (0.369, 0.568) | <0.001 |  | 0.468 (0.369, 0.568) | <0.001 |  |  |  |
